# *Haloimpatiens sporogenes* sp. nov. and *Haloimpatiens myeolchijeotgali* sp. nov., anaerobic bacteria isolated from Myeolchi-jeot, a traditional Korean fermented anchovy

**DOI:** 10.71150/jm.2504009

**Published:** 2025-07-31

**Authors:** Yu Jeong Lee, Byung Hee Chun

**Affiliations:** Department of Microbiology, Pukyong National University, Busan 48513, Republic of Korea

**Keywords:** *Haloimpatiens*, novel species, Myeolchi-jeot, strictly anaerobic

## Abstract

Two rod-shaped, Gram-positive, spore-forming, motile, and strictly anaerobic bacteria, FM7315^T^ and FM7330^T^ were isolated from Myeolchi-jeot, a traditional Korean fermented anchovy. Phylogenetic and phylogenomic analyses based on the 16S rRNA gene and genome sequences revealed that strains FM7315^T^ and FM7330^T^ represent novel species within the genus *Haloimpatiens*. The genome sizes of strains FM7315^T^ and FM7330^T^ were 3,052,517 bp and 4,194,114 bp, respectively, with G + C contents of 29.7 mol% and 28.0 mol%, respectively. Strain FM7315^T^ exhibited growth at 20–37°C, 0–2% NaCl, and pH range of 5.0–8.0, whereas strain FM7330^T^ grew at 25–45°C, 0–4% NaCl, and pH range of 5.0–9.0. Strain FM7315^T^ contains C_14:0_, C_16:0_, C_18:1_ ω9c, Summed Feature 3 (C_16:1_ ω7c/C_16:1_ ω6c), and Summed Feature 8 (C_18:1_ ω7c/C_18:1_ ω6c) as major fatty acids, along with diphosphatidylglycerol, phosphatidylglycerol, glycolipid, two aminophospholipids, and five unidentified lipids. Strain FM7330^T^ contains C_16:0_, C_17:1_ ω8c, and C_18:1_ ω9c as major fatty acids, along with diphosphatidylglycerol, two phosphatidylglycerols, four aminophospholipids, and six unidentified lipids. Based on their phenotypic, chemotaxonomic, and molecular characteristics, strains FM7315^T^ and FM7330^T^ represent two novel species of the genus *Haloimpatiens*, for which the names *Haloimpatiens sporogenes* sp. nov. (FM7315^T^ = KCTC 25939^T^ = JCM 37574^T^) and *Haloimpatiens myeolchijeotgali* sp. nov. (FM7330^T^ = KCTC 25938^T^ = JCM 37575^T^) have been proposed.

## Introduction

The family *Clostridiaceae* encompasses a phylogenetically and phenotypically diverse group of anaerobic spore-forming bacteria. A substantial number of species have been validly published and novel taxa within this family continue to be identified through ongoing taxonomic efforts (Wiegel et al., 2006; Yang et al., 2024). The genus *Haloimpatiens*, which belongs to the family *Clostridiaceae*, was first proposed by Wu et al. (2016). As of this writing, the genus *Haloimpatiens* comprises two species, with one validly published species, *Haloimpatiens lingqiaonensis* isolated from paper mills (Wu et al., 2016), and one invalidly published species, “*Haloimpatiens massiliensis*” isolated from the gut of healthy infants (Anani et al., 2020; Parte et al., 2020). Members of the genus *Haloimpatiens* are strictly anaerobic, rod-shaped, Gram-positive, sensitive to salt stress, and motile with peritrichous flagella. *H. lingqiaonensis*, the type species of the genus *Haloimpatiens*, is distinguished by its cellular polar lipids, including diphosphatidylglycerol (DPG), phosphatidylglycerol (PG), several phospholipids, and glycolipids (GLs), as well as by its major cellular fatty acids, C_14:0_ and C_16:0_. Additionally, the absence of quinones and their reliance on fermentation metabolism results in the production of various fermentation products such as formic acid, acetic acid, ethanol, and lactic acid (Wu et al., 2016). In this study, two novel strains belonging to the genus *Haloimpatiens*, designated as *Haloimpatiens sporogenes* FM7315^T^ and *Haloimpatiens myeolchijeotgali* FM7330^T^, were isolated from Myeolchi-jeot, a traditional Korean fermented anchovy, and their taxonomic characteristics were assessed using a polyphasic approach.

## Materials and Methods

### Isolation and deposition of strains

The strains FM7315^T^ and FM7330^T^ were isolated from Myeolchi-jeot, a traditional Korean fermented anchovy, under anaerobic conditions. Briefly, Myeolchi-jeot samples were purchased from a traditional market in Ganggyeong-eup (36°09'37.3"N, 127°00'56.9"E), Republic of Korea. Following this, all procedures were performed in an anaerobic chamber (COY Laboratory Products) filled with 90% N_2_, 5% CO_2_, and 5% H_2_. The Myeolchi-jeot samples were serially diluted in phosphate-buffered saline, spread onto Bifidobacterium Selective agar (BS; MB cell), and incubated at 30°C for 7 d. After incubation, colonies grown on BS agar were randomly selected based on their morphology and their 16S rRNA genes were PCR-amplified using the universal primers 27F and 1492R (Lane et al., 1991). PCR amplicons were sequenced using the universal primer sets 337F (5’-GACTCCTACGGGAGGCWGCAG-3’), 785F (5’-GGATTAGATACCCTGGTA-3’), and 800R (5’-TACCAGGGTATCTAATCC-3’) (Macrogen, Korea). The sequencing results were assembled into 16S rRNA gene sequences using BioEdit version 7.7.1 (Hall et al., 1999). These sequences were compared with those of all published type species on the EzBioCloud server (www.ezbiocloud.net/identify). Based on this, two novel strains, FM7330^T^ and FM7315^T^, belonging to the family *Clostridiaceae*, were selected for further taxonomic characterization. Strains FM7330^T^ and FM7315^T^ were routinely cultured anaerobically on BS agar at 40°C and 30°C, respectively, for 2 d, and in BL broth (MB Cell) under oxygen-free conditions achieved by nitrogen purging. The strains were preserved at –80°C in a solution containing 10% glycerol (v/v) and 10% skim milk (w/v). Strains FM7315^T^ and FM7330^T^ were deposited in the Korean Collection for Type Cultures (KCTC) and Japan Collection of Microorganisms (JCM) under accession numbers KCTC 25939^T^ and JCM 37574^T^ for FM7315^T^, and KCTC 25938^T^ and JCM 37575^T^ for FM7330^T^. To compare genomic and phenotypic characteristics between strains FM7315^T^ and FM7330^T^, the type strain *Haloimpatiens lingqiaonensis* KCTC 15321^T^ was obtained from the KCTC and was cultivated at 30°C for 2 d on GS agar (KCTC medium no. 511), with the medium prepared according to the KCTC media information.

### 16S rRNA gene-based phylogenetic analysis

The 16S rRNA gene sequences of strains FM7315^T^ and FM7330^T^ with those of closely related type strains were aligned using the SILVA Incremental Aligner (SINA) version 1.2.12 (Pruesse et al., 2012). Phylogenetic trees were then constructed based on neighbor-joining (NJ) (Saitou and Nei, 1987), maximum likelihood (ML) (Felsenstein et al., 1981), and maximum parsimony (MP) (Fitch et al., 1971) algorithms, with 1,000 bootstrap replications, using MEGA11 software (Tamura et al., 2021).

### Whole-genome sequencing and phylogenomic analysis

Genomic DNA of strains FM7315^T^, FM7330^T^, and KCTC 15321^T^ was extracted from cells cultured in optimal media using the standard phenol-chloroform extraction method (Sambrook et al., 1989). The genomic DNA of strain FM7315^T^ was sequenced using the Illumina NovaSeq^TM^ 6000 and PacBio Sequel II systems (Macrogen, Korea). Sequencing reads were assembled using Microbial Genome Analysis software (version SMRT LINK_13.1.0.221970). The genomic DNA of strains FM7330^T^ and KCTC 15321^T^ were sequenced using the Oxford Nanopore MinION platform. Nanopore sequencing reads were de novo-assembled using Flye software (version 2.9.1) (Kolmogorov et al., 2019). The quality of the assembled genomes of the strains FM7315^T^, FM7330^T^, and KCTC 15321^T^ was evaluated based on their completeness and contamination rates using CheckM (version 1.2.2) (Chklovski et al., 2023) with a marker set for the family *Clostridiaceae*. For phylogenomic analysis of strains FM7315^T^ and FM7330^T^, as well as their closely related type strains, the nucleotide sequences of 92 bacterial core genes were obtained from each genome, concatenated, and aligned using the updated bacterial core gene pipeline version 2 (UBCG2) (Kim et al., 2021). The concatenated alignment was then used to infer a ML phylogenomic tree with 1,000 bootstrap replications using MEGA11 software (Tamura et al., 2021).

To determine the genus-level affiliation of strains FM7315^T^ and FM7330^T^, genome-based analyses of average amino acid identity (AAI; https://github.com/endixk/ezaai) and percentage of conserved proteins (POCP; https://github.com/hoelzer/pocp) were performed using the genomes of strains FM7315^T^, FM7330^T^, and closely related type strains from the genera *Haloimpatiens* (*H. lingqiaonensis* KCTC 15321^T^), and *Clostridium* (*C. butyricum* DSM 10702^T^). For species-level comparisons, the average nucleotide identity (ANI) (Lee et al., 2016) and digital DNA-DNA hybridization (dDDH) values were calculated against the closest type strain, *H. lingqiaonensis* KCTC 15321^T^. The dDDH values were obtained using the Genome-to-Genome Distance Calculator (GGDC; https://ggdc.dsmz.de/) based on formula 2 with the recommended BLAST+ method (Meier-Kolthoff et al., 2013).

### Genome annotation and analysis

The whole-genome sequences of strains FM7315^T^, FM7330^T^, and KCTC 15321^T^ were annotated using the NCBI Prokaryotic Genome Automatic Annotation Pipeline version 6.7 (Tatusova et al., 2016). The complete genomes of the strains FM7315^T^, FM7330^T^, and KCTC 15321^T^ were visualized using Proksee (Grant et al., 2023). Functional genes were predicted based on the protein-coding sequences in the KEGG database (Kanehisa and Goto, 2000). The carbohydrate-active enzyme (CAZy) genes in the genomes of strains FM7315^T^, FM7330^T^, and KCTC 15321^T^ were identified using the dbCAN3 meta server (https://bcb.unl.edu/dbCAN2/) with default parameters.

### Physiological and morphological analyses

Strains FM7315^T^ and FM7330^T^ were tested for their ability to grow on BS agar, Brain Heart Infusion agar (BHI; MB Cell), Yeast extract Peptone Dextrose agar (YPD; MB Cell), and Reasoner’s 2A agar (R2A; Difco) at 30°C for 7 d under anaerobic conditions. The growth conditions of strains FM7315^T^ and FM7330^T^ were evaluated on BS agar at various temperatures (4–50°C), and in BL broth with different NaCl concentrations (0–10%, w/v) and pH values (4.0–10.0), all tested for 7 d under anaerobic conditions. BL broth of pH below 8.0 and pH 8.0–9.5 were prepared using Na_2_HPO_4_/NaH_2_PO_4_ and Tris/HCl buffers, respectively (Gomori, 1995). After sterilization (121°C for 15 min), the pH values were adjusted again, if necessary. Growth of strains FM7315^T^ and FM7330^T^ under aerobic condition was evaluated on BS agar at their optimal growth temperature for 7 d. Cell morphology of strains FM7315^T^ and FM7330^T^ was examined using field emission scanning electron microscopy (FE-SEM; JSM-6700F, JEOL). Motility was determined using semi-solid BL agar (0.4% agar) by stab inoculation (Tittsler and Sandholzer, 1936). Gram staining was performed as described by Gram (1884), and spore formation was examined by endospore staining of heat-treated cells (80°C for 20 min) (Reynolds et al., 2009). The oxidase and catalase activities were tested using an oxidase reagent (bioMérieux) and 3% hydrogen peroxide (Junsei), respectively. Hydrolytic activities of strains FM7315^T^ and FM7330^T^ were evaluated on BS agar supplemented with 6% (w/v) skim milk, starch, Tween 20, and Tween 80 (Gerhardt et al., 1994). The substrate utilization profiles of strains FM7315^T^ and FM7330^T^, diverse sugars (20 mM), alcohols (20 mM), organic acids (20 mM), and amino acids (10 mM), were evaluated in minimal basal medium (Hernández-Eugenio et al., 2002). Fermentation products of strains FM7315^T^ and FM7330^T^ were analyzed using culture supernatants obtained after 3 days of incubation in BL broth under optimal growth conditions. Organic acids and alcohols were subsequently analyzed using liquid chromatography–tandem mass spectrometry with triple quadrupole and gas chromatography–mass spectrometry, respectively, according to Agilent Technologies (2016) and Lee et al. (2013). Biochemical properties were assessed using an API ZYM kit (bioMérieux) according to the manufacturer’s instructions, with all procedures performed under strict anaerobic conditions.

### Chemotaxonomic characterization

For cellular fatty acid analysis, strains FM7315^T^ and FM7330^T^ were cultivated on BL medium, while *Haloimpatiens lingqiaonensis* KCTC 15321^T^ was grown on GS medium under their respective optimal growth conditions. Cellular fatty acids were extracted using the standard MIDI protocol (Sherlock Microbial Identification System, version 6.0B) and analyzed using a gas chromatograph (Hewlett-Packard 6890) with reference to the TSBA6 database (Sasser, 1990). For polar lipid analysis, strains FM7315^T^ and FM7330^T^ were cultivated on BL medium, under their respective optimal growth conditions. Polar lipids were extracted using the chloroform–methanol method and subsequently analyzed via two-dimensional thin-layer chromatography, as described by Minnikin et al. (1977). The polar lipid composition was determined using four spray reagents: 10% ethanolic molybdophosphoric acid for total polar lipids, ninhydrin for aminolipids, Dittmer-Lester reagent for phospholipids, and *α*-naphthol and sulfuric acid for GLs.

## Results and Discussion

### 16S rRNA gene-based phylogenetic analysis

**Fig. 1.**
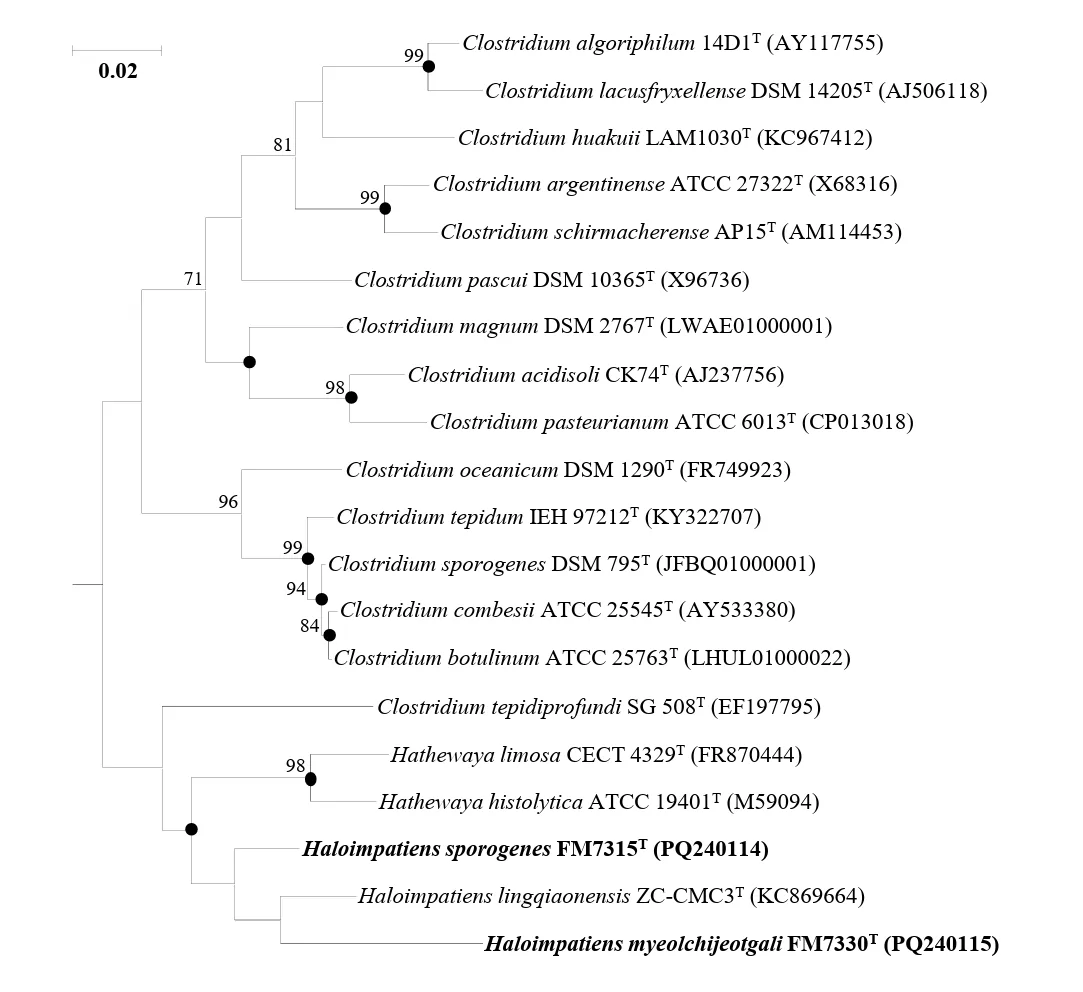
A maximum-likelihood tree based on 16S rRNA gene sequences showing the phylogenetic relationships of strains *H. sporogenes* FM7315^T^, *H. myeolchijeotgali* FM7330^T^, and their closely related taxa. Bootstrap values with more than 70% are shown on the nodes as percentages of 1,000 replicates. Filled circles (●) indicate nodes that were also identified in trees reconstructed with the neighbor-joining and maximum-likelihood algorithms. *Bacillus subtilis* NCIB 3610^T^ (ABQL01000001) was selected as the outgroup. The scale bar, 50 changes per nucleotide position.

Analysis of the 16S rRNA gene sequence identities revealed that strains FM7315^T^ and FM7330^T^ were phylogenetically related to *Haloimpatiens lingqiaonensis* ZC-CMC3^T^, with 16S rRNA gene sequence similarities of 96.58% and 94.14%, respectively. The identity between strains FM7315^T^ and FM7330^T^ was 94.33%, all of which are significantly below the species threshold of 98.7% (Chun et al., 2018). Phylogenetic analysis based on the ML algorithm showed that strains FM7315^T^ and FM7330^T^ formed a distinct lineage, clustering with *H. lingqiaonensis* ZC-CMC3^T^ but supported by a low bootstrap value (Fig. 1). Phylogenetic trees reconstructed using NJ and MP methods showed that FM7315^T^ and FM7330^T^ formed a distinct clade with *H. lingqiaonensis* ZC-CMC3^T^ (Fig. S1).

### Genome characteristics

The complete genomes of the strains FM7315^T^ and FM7330^T^ and the reference strain KCTC 15321^T^ were obtained using genome sequencing and assembly (Fig. S2). The genome of strain FM7315^T^ comprised one complete chromosome and one circular plasmid, whereas that of strain FM7330^T^ included one complete chromosome and two circular plasmids. In contrast, the genome of strain KCTC 15321^T^ contained only one complete chromosome. Genome quality assessment of strains FM7315^T^, FM7330^T^, and KCTC 15321^T^ using CheckM (version 1.2.2) revealed completeness values of 99.36%, 98.31%, and 92.53%, respectively, highlighting the high quality and completeness of their genomes (Chklovski et al., 2023).

**Fig. 2.**
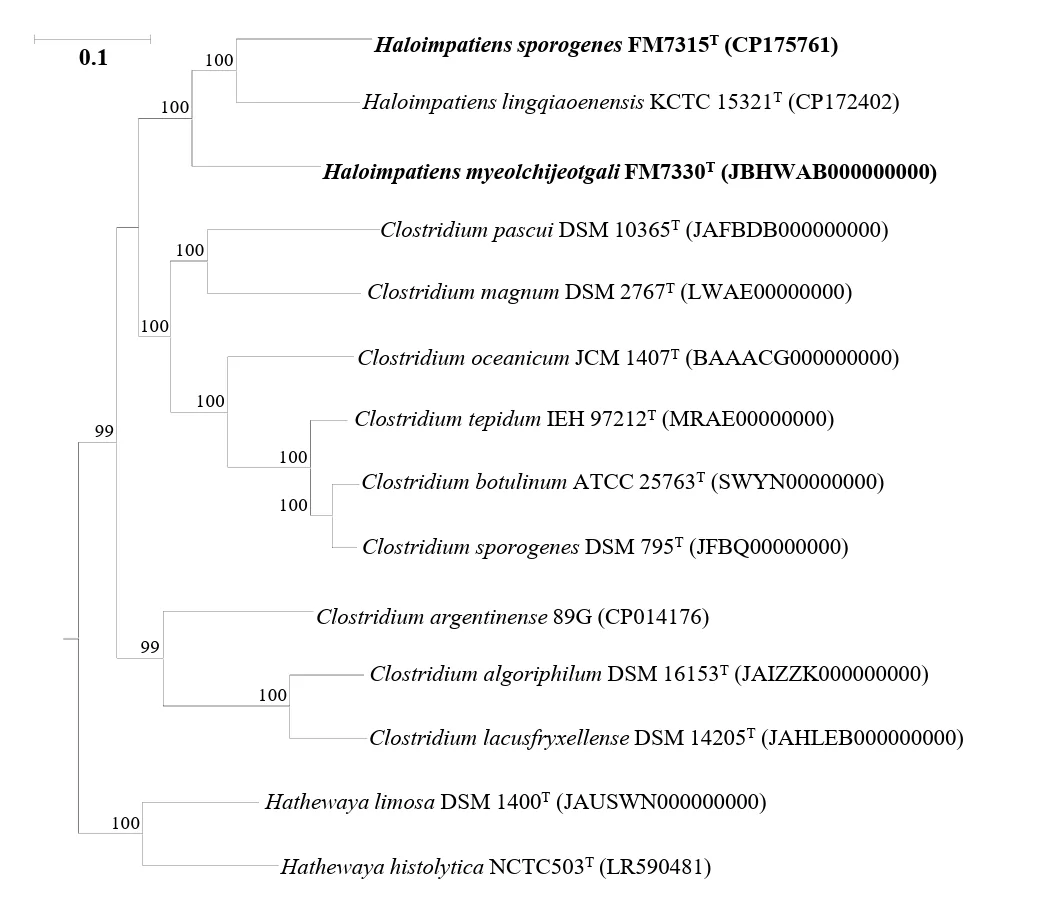
A maximum-likelihood tree showing the phylogenomic relationships of strains *H. sporogenes* FM7315^T^ (CP175761), *H. myeolchijeotgali* FM7330^T^ (JBHWAB000000000), and related taxa, based on concatenated 92 core gene sequences. Bootstrap values with more than 70% are shown on the nodes as percentages of 1,000 replicates. *Bacillus subtilis* DSM 10^T^ (JAEPVU000000000) was selected as the outgroup. The scale bar, 0.1 changes per nucleotide position.

Genome-based phylogenomic analysis showed that strains FM7315^T^ and FM7330^T^ formed a distinct clade with *H. lingqiaonensis* KCTC 15321^T^ (Fig. 2). To clarify their taxonomic positions at the genus level, AAI and POCP were analyzed (Table S1). Strains FM7315^T^ and FM7330^T^ showed high genomic identities with *H. lingqiaonensis* KCTC 15321^T^, which were above the genus delineation thresholds of 60–65% for AAI (Riesco and Trujillo, 2024) and 50% for POCP (Qin et al., 2014). These results supported the classification of FM7315^T^ and FM7330^T^ within the genus *Haloimpatiens*. To assess species-level relatedness, the ANI and dDDH values were calculated for strains FM7315^T^, FM7330^T^, and *H. lingqiaonensis* KCTC 15321^T^ (Table S2). The ANI values between FM7315^T^ and FM7330^T^ and *H. lingqiaonensis* KCTC 15321^T^ were 74.64% and 74.27%, respectively, which are below the species delineation threshold of 95–96% (Lee et al., 2016). Similarly, the dDDH values for the same pairs were 22.60% and 22.10%, respectively, both of which are below the 70% threshold for species delineation (Meier-Kolthoff et al., 2013). These results indicated that strains FM7315^T^ and FM7330^T^ represent novel species within the genus *Haloimpatiens*.

**Fig. 3.**
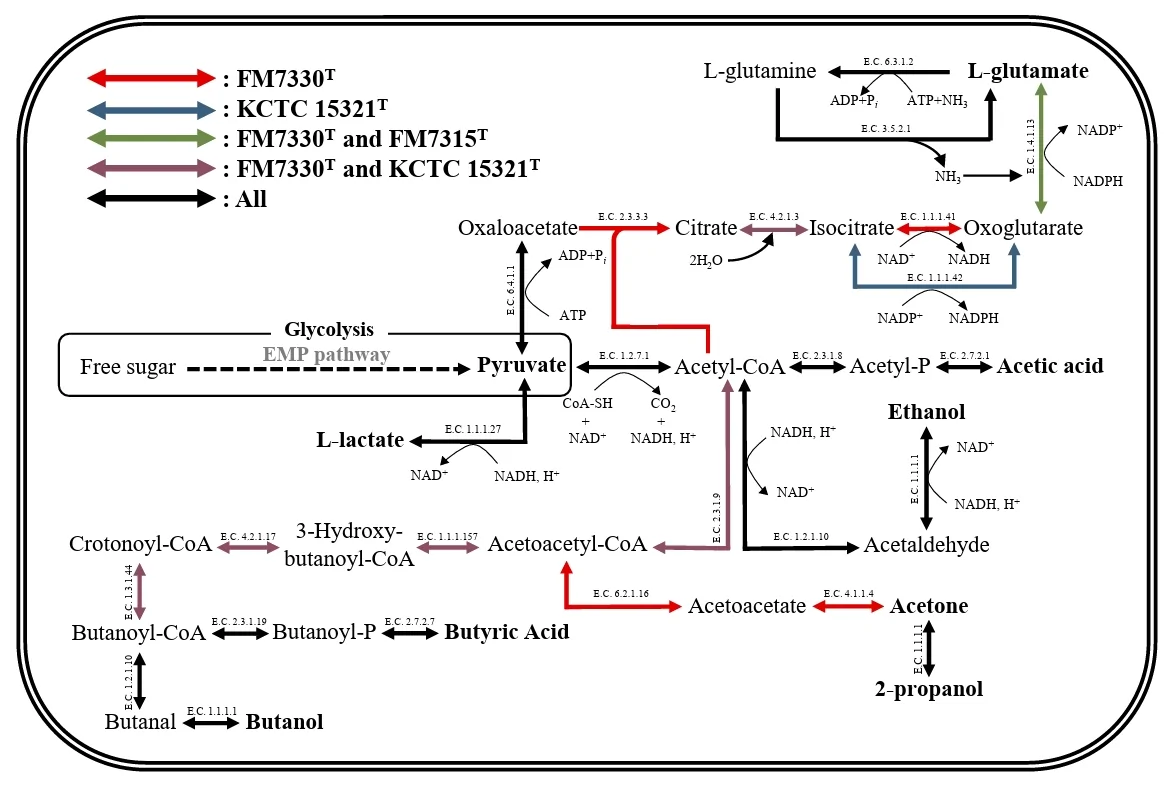
Carbon metabolic pathways of the strains FM7315^T^, FM7330^T^, and KCTC 15321^T^ for fermentation. The arrow colors indicate the metabolic pathways as follows: red represents pathways exclusive to FM7330^T^, blue represents those unique to KCTC 15321^T^, green represents pathways shared between FM7330^T^ and FM7315^T^, purple represents those common to FM7330^T^ and KCTC 15321^T^, and black represents pathways present in all three strains.

The genome sizes of strains FM7315^T^, FM7330^T^, and KCTC 15321^T^ are 3.1, 4.2, and 3.3 Mbp, respectively, with G + C contents of 29.5%, 28.0%, and 31.0%, respectively. These values are consistent with the genome sizes and G + C contents of species within the genus *Haloimpatiens* currently registered in the GenBank database (3.3–4.2 Mbp and 30–31%). The genomes of strains FM7315^T^, FM7330^T^, and KCTC 15321^T^ harbored 2,893, 3,769, and 3,182 genes, respectively, including 2,728, 3,581, and 2,936 protein-coding genes; 93, 94, and 91 tRNA genes; and 30, 31, and 27 rRNA genes, respectively. The genomes of strains FM7315^T^, FM7330^T^, and KCTC 15321^T^ were predicted to contain 43, 71, and 43 genes, respectively, encoding various CAZy. Strain FM7330^T^ had a higher ability to degrade polysaccharides than strains FM7315^T^ and KCTC 15321^T^. Especially, strain FM7330^T^ uniquely possesses genes involved in 6 glycoside hydrolase families (EC 3.2.1.-), two glycosyltransferase families (EC 2.4.1.-), two auxiliary activity families (EC 1.1.3.- and EC 1.11.1.-), and two carbohydrate-binding module families, which contribute to their ability to degrade diverse carbohydrates and support their metabolic functions during food fermentation. KEGG analysis revealed that strains FM7315^T^, FM7330^T^, and KCTC 15321^T^ contained genes associated with lactic acid and ethanol production, suggesting their potential role in fermentation-related metabolic processes under anaerobic conditions (Fig. 3). In addition, metabolic genes related to butyric acid production were identified in the genomes of strains FM7330^T^ and KCTC 15321^T^, while strain FM7315^T^ was found to possess only a subset of the associated genes. Nevertheless, butyric acid was detected in all three strains, suggesting that strain FM7315^T^ may harbor alternative genes or pathways involved in its biosynthesis. Strains FM7315^T^ and FM7330^T^ contained a gene encoding glutamate dehydrogenase, an enzyme important for glutamate production. Glutamate is a key compound that gives food an umami flavor. This suggests that strains FM7315^T^ and FM7330^T^ may enhance umami taste during food fermentation. Moreover, KEGG pathway analysis identified genes related to flagellar assembly and motility in the genomes of FM7315^T^ and FM7330^T^ (Table S3).

### Physiological and morphological characteristics

Strains FM7315^T^ and FM7330^T^ grew well on BS agar, BHI agar, and YPD agar but did not grow on R2A agar. The growth conditions for strain FM7315^T^ were exhibited within a temperature range of 20–37°C (optimal 30°C), NaCl concentration range of 0–2% (optimal 0%), and pH range of 5.0–8.0 (optimal 6.5). In contrast, strain FM7330^T^ exhibited slightly different growth conditions, with a temperature range of 25–45°C (optimal 40°C), NaCl concentration range of 0–4% (optimal 2%), and pH range of 5.0–9.0 (optimal 7.0). Strains FM7315^T^ and FM7330^T^ are Gram-positive and spore forming. Cells observed under an electron microscope were rod-shaped, measuring 1.7–5.7 µm in length and 0.4–0.9 µm in width for strain FM7315^T^, and 2.1–7.4 µm in length and 0.7–2.1 µm in width for strain FM7330^T^. Motility tests on semi-solid BL agar confirmed that both FM7315^T^ and FM7330^T^ were motile. Aerobic growth was not observed after 21 d of incubation under optimal conditions.

Strains FM7315^T^ and FM7330^T^ shared several phenotypic traits with *Haloimpatiens lingqiaonensis* KCTC 15321^T^, such as catalase and oxidase activity and the inability to hydrolyze Tween 20 and Tween 80. However, the three strains exhibited distinct characteristics (Table 3)[Table t1-jm-2504009][Table t2-jm-2504009]. Strains FM7315^T^ and FM7330^T^ hydrolyzed starch but not casein, whereas strain KCTC 15321^T^ hydrolyzed casein but not starch. API ZYM testing revealed that all three strains were positive for acid phosphatase and naphthol-AS-BI phosphohydrolase. Strain FM7330^T^ exhibited esterase (C4) and esterase lipase (C8) activity. Strain FM7315^T^ utilizes D-fructose, D-glucose, and D-maltose, whereas strain FM7330^T^ metabolizes D-ribose and pyruvate. These results were further supported by the identification of relevant metabolic genes in the genomes of both strains, particularly by the observation that strain FM7330^T^ harbored a greater number of CAZy families, consistent with its broader substrate utilization profile. Butyrate was uniquely produced by strains FM7315^T^ and FM7330^T^, whereas it was not detected in strain ZC-CMC3^T^. Additionally, strains FM7315^T^ and ZC-CMC3^T^ produced ethanol, whereas strain FM7330^T^ did not.

### Chemotaxonomic characteristics

The polar lipid and cellular fatty acid profiles of strains FM7315^T^ and FM7330^T^ differed from those of *Haloimpatiens lingqiaonensis* KCTC 15321^T^. Cellular fatty acid analysis revealed that C_16:0_ was abundant in all three strains (Table S4). In contrast, C_14:0_ was detected only in FM7315^T^ and KCTC 15321^T^. FM7315^T^ and FM7330^T^ shared C_18:1_ ω9c as a major component, whereas this fatty acid was not prominent in KCTC 15321^T^. Additionally, FM7315^T^ possessed Summed Feature 3 (C_16:1_ ω7c/C_16:1_ ω6c) and Summed Feature 8 (C_18:1_ ω7c/C_18:1_ ω6c) as major components, whereas FM7330^T^ contained C_17:1_ ω8c. Strains FM7315^T^ and FM7330^T^ both harbor two major polar lipids, DPG and PG. In addition, strains FM7315^T^ and FM7330^T^ shared five unidentified polar lipids (ULs) and two aminophospholipids (APLs) not observed in strain ZC-CMC3^T^. Strain FM7315^T^ also possessed an additional GL, whereas FM7330^T^ contained two additional APLs. These compositional differences are common within the family *Clostridiaceae* (Chaikitkaew et al., 2022; Flaiz et al., 2020; Xu et al., 2019), and are presumed to be influenced by environmental factors related to the isolation of strains. These variations are likely attributable to the environmental factors associated with the respective sources of isolation.

### Taxonomic Conclusion

#### Description of *Haloimpatiens sporogenes* sp. nov.

***Haloimpatiens sporogenes*** (spo.ro’ge.nes. Gr. fem. n. spora, a spore; Gr. v. gennao, produce; and N.L. masc. part. adj. sporogenes, spore-producing). Cells are strictly anaerobic, spore forming, Gram-positive, motile, rod-shaped, 1.7–5.7 µm in length, and 0.4–0.9 µm in width. Growth is observed on BS agar at temperatures of 20–37°C (optimum 30°C), NaCl concentrations of 0–2% (optimum 0%), and with a pH range of 5.0–8.0 (optimum 6.5). Cells hydrolyze starch; assimilate D-fructose, D-glucose, and D-maltose as sole carbon sources; and exhibit positive enzymatic activity for acid phosphatase and naphthol-AS-B1 phosphohydrolase. Acetic, butyric, lactic acids, and ethanol were the major fermentation products. The major fatty acids are C_14:0_, C_16:0_, C_18:1_ ω9c, Summed Feature 3 (C_16:1_ ω7c/C_16:1_ ω6c), and Summed Feature 8 (C_18:1_ ω7c/C_18:1_ ω6c), while the major polar lipids are diphosphatidylglycerol, phosphatidylglycerol, glycolipid, aminophospholipids and unidentified lipids. The type strain is FM7315^T^ (= KCTC 25939^T^ = JCM 37574^T^), isolated from Myeolchi-jeot, a traditional Korean fermented anchovy. The total genome size, including both the chromosome and the plasmid, is 3,052,517 bp, with a G + C content of 29.7 mol%. The GenBank accession numbers for the 16S rRNA gene and complete genome sequences are PQ240114 and CP175761, respectively.

#### Description of *Haloimpatiens myeolchijeotgali* sp. nov.

***Haloimpatiens myeolchijeotgali*** (mye.ol.chi.je.ot'ga.li. N.L. gen. n. myeolchijeotgali, Myeolchi-jeotgal, traditional Korean fermented anchovy). Cells are strictly anaerobic, spore forming, Gram-positive, motile, rod-shaped, 2.1–6.3 µm in length and 0.7–2.1 µm in width. Growth is observed on BS agar at temperatures of 25–45°C (optimum 40°C), NaCl concentration of 0–4% (optimum 2%), and with a pH range of 5.0–9.0 (optimum 7.0). Cells hydrolyze starch; assimilate pyruvate, D-fructose, D-glucose, D-maltose, and D-ribose as sole carbon sources; and exhibit positive enzymatic activity for acid phosphatase, naphthol-AS-B1 phosphohydrolase, esterase, and esterase lipase. Acetic, butyric, and lactic acids are the major fermentation products. The major fatty acids are C_16:0_, C_17:1_ ω8c, and C_18:1_ ω9c, while the major polar lipids are diphosphatidylglycerol, phosphatidylglycerol, aminophospholipids, and unidentified lipids. The type strain is FM7330^T^ (= KCTC 25938^T^ = JCM 37575^T^), isolated from Myeolchi-jeot, a traditional Korean fermented anchovy. The total genome size, comprising one chromosome and two plasmids, is 4,194,114 bp, with a G + C content of 28.0 mol%. The GenBank accession numbers for the 16S rRNA gene and complete genome sequences are PQ240115 and JBHWAB000000000, respectively.

## Figures and Tables

**Table 1. t1-jm-2504009:** Genomic features of the two novel strains and *Haloimpatiens lingqiaonensis* KCTC 15321^T^. Strains; 1, *H. sporogenes* FM7315^T^ (this study); 2, *H. myeolchijeotgali* FM7330^T^ (this study); 3, *H. lingqiaonensis* KCTC 15321^T^ (this study)

Feature	1	2	3
Genome size (Mb)	3.0	4.2	3.5
Status	Complete	Complete	Complete
No. of contigs	2	3	1
Chromosome	1	1	1
Plasmid	1	2	0
G + C contents (mol%)	29.5	28.0	31.0
N50	3.0 Mb	4.0 Mb	3.5 Mb
No. of total genes	2,893	3,769	3,182
No. of protein-coding genes	2,728	3,581	2,936
No. of total RNA genes	127	129	123
No. of tRNA genes	93	94	91
No. of rRNA genes	30	31	27
No. of total CAZy genes	43	71	43
Glycoside hydrolase	12	22	10
Glycosyltransferase	20	29	23
Carbohydrate esterase	7	10	6
Auxiliary activity	1	8	0
Carbohydrate-binding module	3	2	4
No. of ncRNA genes	4	4	5
No. of pseudogenes	38	59	123
Coverage (×)	175	114	65
GenBank accession NO.	CP175761	JBHWAB000000000	CP172402

**Table 2. t2-jm-2504009:** Comparison of phenotypic characteristics between the two novel strains and *Haloimpatiens lingqiaonensis* KCTC 15321^T^. Strains; 1, *H. sporogenes* FM7315^T^ (this study); 2, *H. myeolchijeotgali* FM7330^T^ (this study); 3, *H. lingqiaonensis* KCTC 15321^T^ (this study). All strains were positive for the following: enzyme activities of acid phosphatase and naphthol-AS-B1 phosphohyderolase. All strains were negative for the following: hydrolysis of Tween 20 and Tween 80; enzyme activities of leucine arylamidase, *α*-chymotrypsin, lipase, valine arylamidase, cystine arylamidase, trypsin, *α*-galactosidase, *β*-galactosidase, *β*-glucuronidase, *α*-glucosidase, *β*-glucosidase, N-acetyl-*β*-glucosaminidase, *α*-mannosidase, catalase and oxidase; and utilization of L-arabinose, D-galactose, D-mannitol, D-xylose, L-xylose, formic acid, acetic acid, butyric acid, propionic acid, fumaric acid, lactic acid, malic acid, succinic acid, ethanol, butanol, propanol, arginine, asparagine, aspartate, glutamine, glycine, tryptophan, tyrosine, alanine, histidine, isoleucine, lysine, methionine, phenylalanine, serine, threonine, and valine as single carbon sources. +, Positive; –, negative. A, acetate; B, butyrate; E, ethanol; F, formate; L, lactate

Characteristics	1	2	3
Isolation Source^†^	Myeolchi-jeot	Myeolchi-jeot	Paper-mill wastewater
Growth range^†^ of:			
Temperature (optimum, ℃)	20–37 (30)	25–45 (40)	25–48 (43)
pH (optimum)	5.0–8.0 (6.5)	5.0–9.0 (7)	5.5–8.0 (7)
NaCl (optimum, %)	0–2 (0)	0–4 (2)	0–3 (0)
Spore forming^†^	+	+	–
Hydrolysis^†^ of:			
Starch	+	+	–
Casein	–	–	+
Enzyme activities (API ZYM):			
Alkaline phosphate	–	–	+
Esterase (C4)	–	+	–
Esterase lipase (C8)	–	+	–
α-Fucosidase		–	+
Utilization^†^ of:			
Pyruvate	-	+	–
D-Fructose	+	+	–
D-Glucose	+	+	–
D-Maltose	+	+	–
D-Ribose	–	+	–
Fermentation products^†*^	A, B, E, L	A, B, L	F, A, E, L

† The results for *H. lingqiaonensis* were obtained from a previous study on *H. lingqiaonensis* ZC-CMC3^T^ (Wu et al., 2016).

* Fermentation product analysis of *H. lingqiaonensis* ZC-CMC3^T^ was performed in PYG medium (Wu et al., 2016).

## Supplementary Information

Table S1. Comparison of the genomic identities of strains FM7315^T^ and FM7330^T^ with those of their phylogenetically closely related species in the genus *Haloimpatiens* and the type species of the genus *Clostridium*, using AAI and POCP analysis. Strains: 1, *Haloimpatiens sporogenes* FM7315^T^; 2, *Haloimpatiens myeolchijeotgali* FM7330^T^; 3, *Haloimpatiens lingqiaonensis* KCTC 15321^T^; 4, *Clostridium butyricum* DSM 10702^T^

Table S2. Pairwise comparison of average nucleotide identity (ANI) and digital DNA-DNA hybridization (dDDH) values between strains FM7315^T^ and FM7330^T^ and the type strain of phylogenetically closely related species in the genus *Haloimpatiens*. Strains: 1, *Haloimpatiens sporogenes* FM7315^T^; 2, *Haloimpatiens myeolchijeotgali* FM7330^T^; 3, *Haloimpatiens lingqiaonensis* KCTC 15321^T^

Table S3. Flagellar biosynthesis-related genes identified in the genomes of strains FM7315ᵀ and FM7330ᵀ

Table S4. Comparison of cellular fatty acid compositions (%) between the two novel strains and *Haloimpatiens lingqiaonensis* KCTC 15321^T^. Strains: 1, *H. sporogenes* FM7315^T^, 2, FM7330^T^, 3, *H. lingqiaonensis* KCTC 15321^T^. The majority of fatty acid values (> 5%) are highlighted in bold. tr, trace element (< 1%); –, not detected

Fig. S1. Neighbor-joining (A) and maximum-parsimony (B) trees based on 16S rRNA gene sequences showing the phylogenetic relationships of strains *H. sporogenes* FM7315^T^, *H. myeochijeotgali* FM7330^T^, and their closely related taxa. Bootstrap values with more than 70% are shown on the nodes as percentages of 1,000 replicates. *Bacillus subtilis* NCIB 3610^T^ (ABQL01000001) was selected as the outgroup.

Fig. S2. Genome maps for strains *H. sporogenes* FM7315^T^ (A), *H. myeolchijeotgali* FM7330^T^ (B), and *H. lingqiaonensis* KCTC 15321^T^ (C).

Fig. S3. Scanning electron micrograph of strains FM7315^T^ (A) and FM7330^T^ (B) cells. Bar, μm.

Fig. S4. Two-dimensional thin-layer chromatoGrams showing the compositions of total polar lipids (A), glycolipids (B), aminolipids (C), and phospholipids (D) in strains FM7315^T^. Total lipids were stained with 90% ethanolic molybdophosphoric acid, glycolipids with α-naphthol and sulfuric acid, amino lipids with ninhydrin, and phospholipids with both ninhydrin and the Dittmer-Lester reagent. Solvent system: (I) chloroform-methanol-water (65:25:4, v/v/v); (II) chloroform-acetic acid-methanol-water (80:15:12:4, v/v/v/v). DPG, diphosphatidylglycerol; PG, phosphatidylglycerol; APL, aminophospholipid; GL, unidentified glycolipid; UL, unidentified polar lipid.

Fig. S5. Two-dimensional thin-layer chromatoGrams showing the compositions of total polar lipids (A), glycolipids (B), aminolipids (C), and phospholipids (D) in strains FM7330^T^. Total lipids were stained with 90% ethanolic molybdophosphoric acid, glycolipids with α-naphthol and sulfuric acid, amino lipids with ninhydrin, and phospholipids with both ninhydrin and the Dittmer-Lester reagent. Solvent system: (I) chloroform-methanol-water (65:25:4, v/v/v); (II) chloroform-acetic acid-methanol-water (80:15:12:4, v/v/v/v). DPG, diphosphatidylglycerol; PG, phosphatidylglycerol; APL, aminophospholipid; UL, unidentified polar lipid.
