# Supplementary material for: Haloimpatiens sporogenes sp. nov. and Haloimpatiens myeolchijeotgali sp. nov., anaerobic bacteria isolated from Myeolchi-jeot, a traditional Korean fermented anchovy
Source: J Microbiol. 2025 Jul 31;63(7):e2504009. doi: 10.71150/jm.2504009 (PMC13577056; doi:10.71150/jm.2504009)

Fig. S1. Neighbor-joining (A) and maximum-parsimony (B) trees based on 16S rRNA gene sequences showing the phylogenetic relationships of strains *H. sporogenes* FM7315^{T}, *H. myeochijeotgali* FM7330^{T}, and their closely related taxa. Bootstrap values with more than 70% are shown on the nodes as percentages of 1,000 replicates. *Bacillus subtilis* NCIB 3610^{T} (ABQL01000001) was selected as the outgroup.

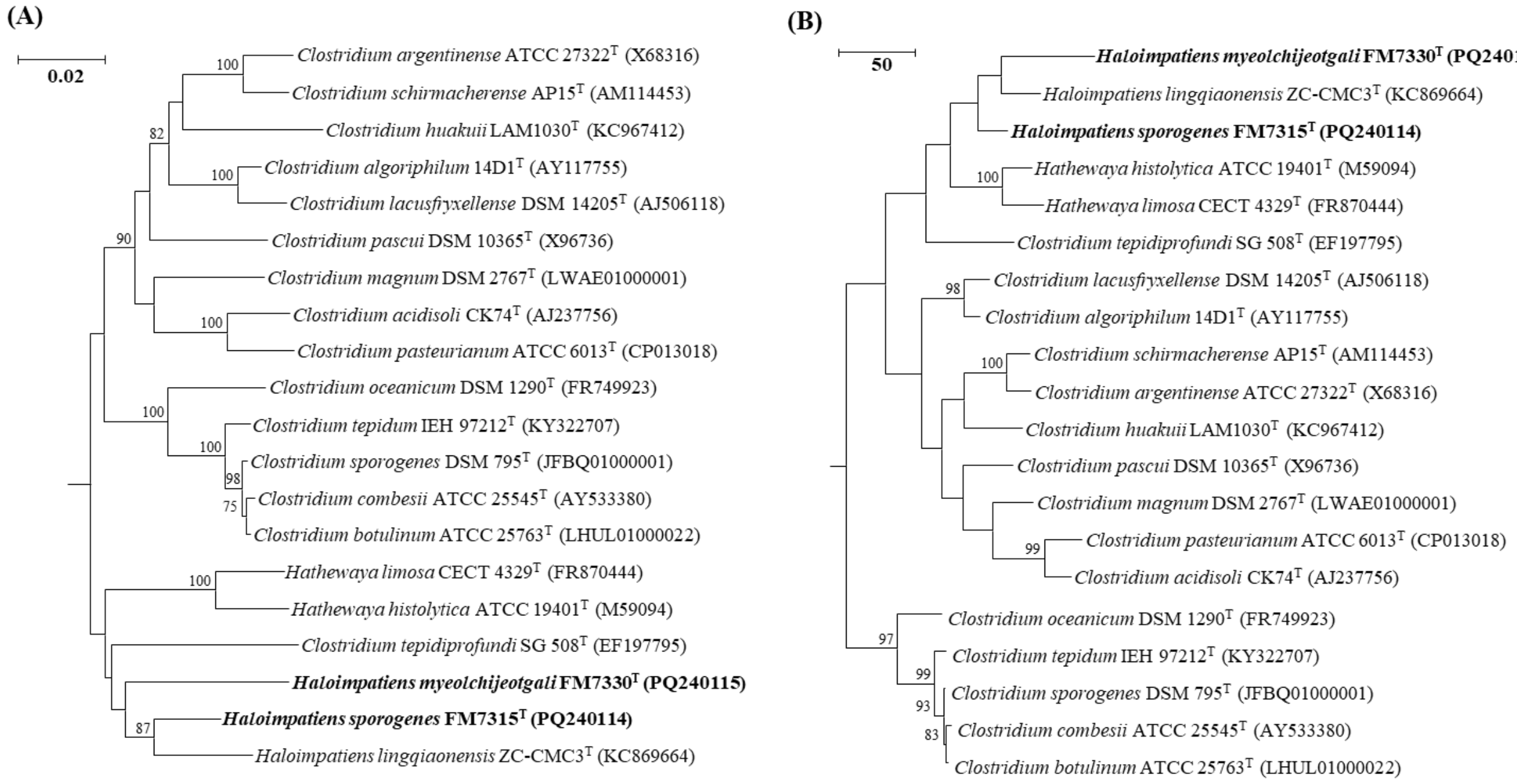

Supplement: Supplementary file 1 [file jm-2504009-Supplementary-Fig-S1.pdf]
