# Supplementary material for: Haloimpatiens sporogenes sp. nov. and Haloimpatiens myeolchijeotgali sp. nov., anaerobic bacteria isolated from Myeolchi-jeot, a traditional Korean fermented anchovy
Source: J Microbiol. 2025 Jul 31;63(7):e2504009. doi: 10.71150/jm.2504009 (PMC13577056; doi:10.71150/jm.2504009)

Fig. S2. Genome maps for strains *H. sporogenes* FM7315^{T} (A), *H. myeolchijeotgali* FM7330^{T} (B), and *H. lingqiaonensis* KCTC 15321^{T} (C).

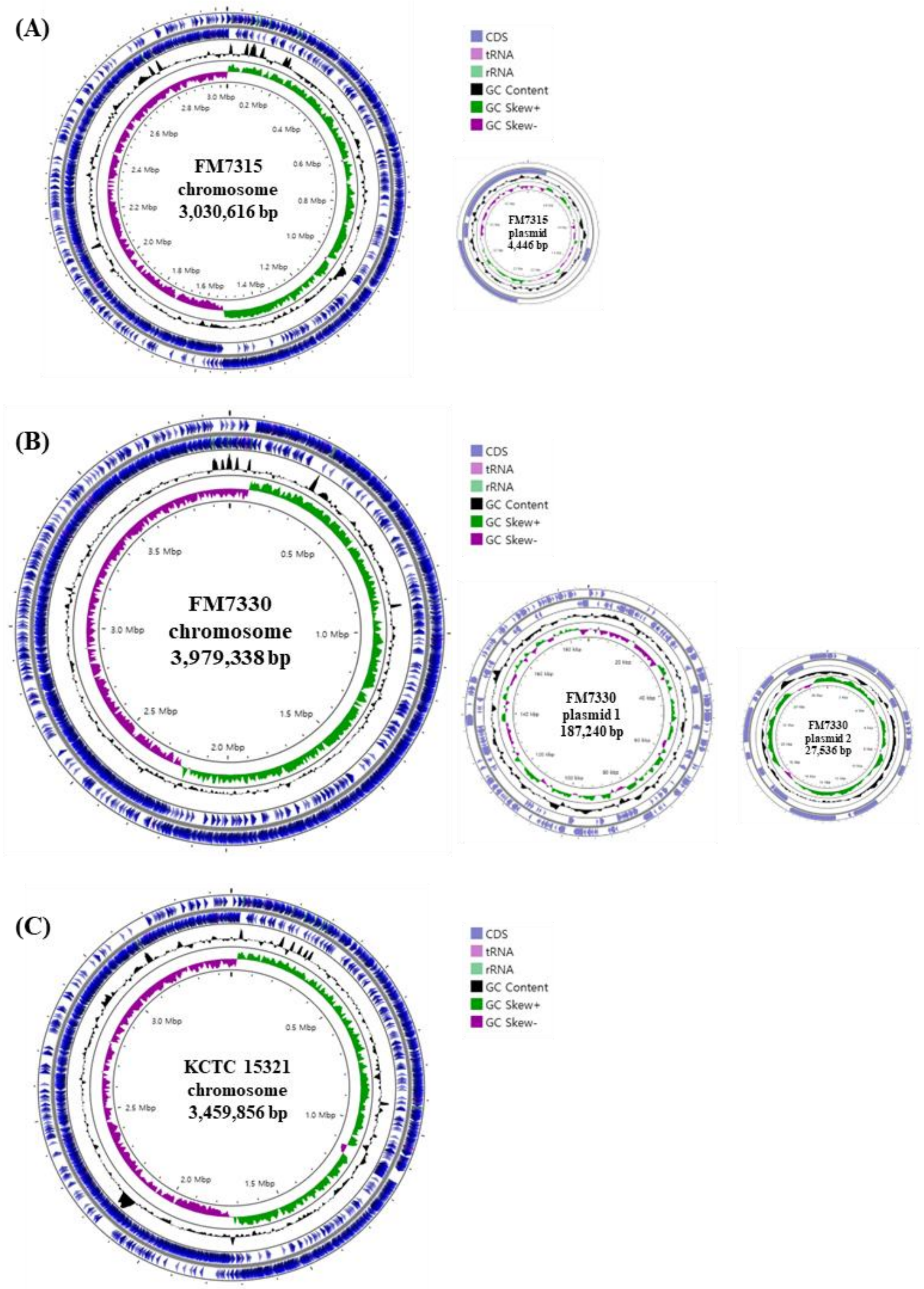

Supplement: Supplementary file 2 [file jm-2504009-Supplementary-Fig-S2.pdf]
