# Supplementary material for: Haloimpatiens sporogenes sp. nov. and Haloimpatiens myeolchijeotgali sp. nov., anaerobic bacteria isolated from Myeolchi-jeot, a traditional Korean fermented anchovy
Source: J Microbiol. 2025 Jul 31;63(7):e2504009. doi: 10.71150/jm.2504009 (PMC13577056; doi:10.71150/jm.2504009)

Fig. S3. Scanning electron micrograph of strains FM7315^{T} (A) and FM7330^{T} (B) cells. Bar, μm.

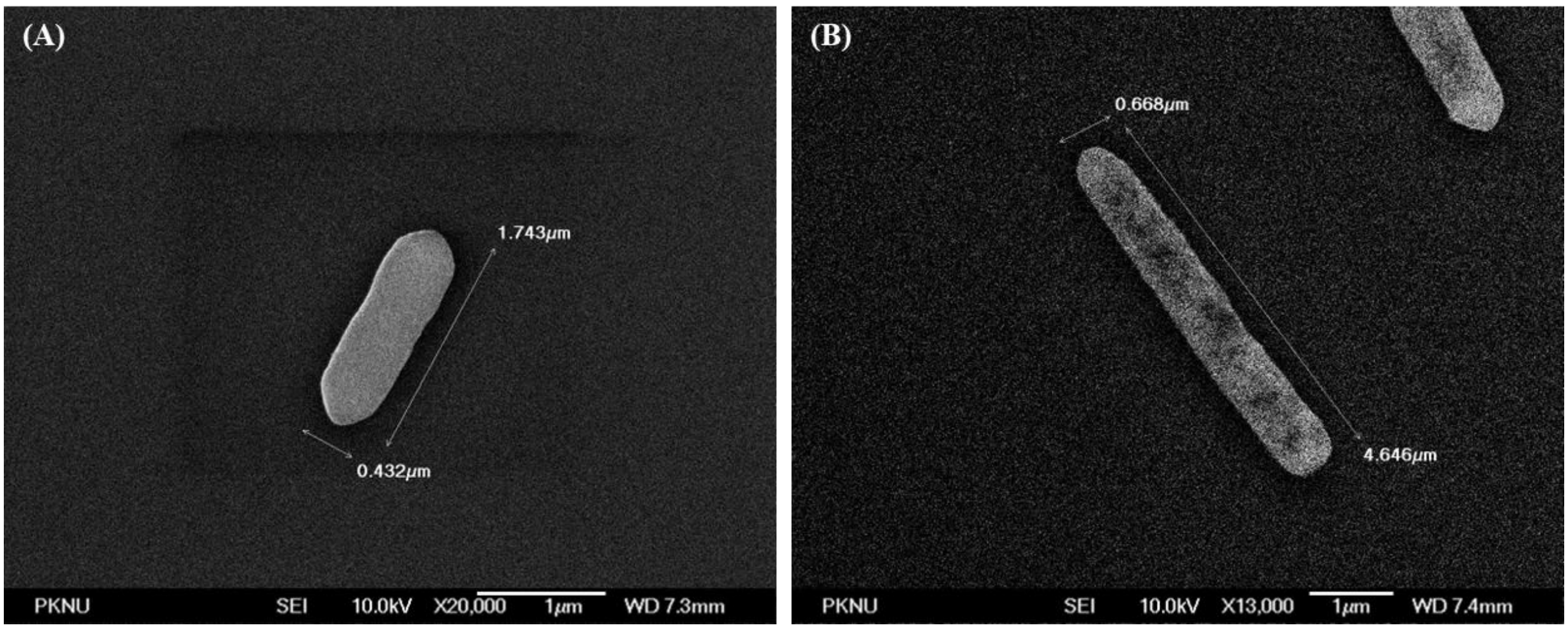

Supplement: Supplementary file 3 [file jm-2504009-Supplementary-Fig-S3.pdf]
