# Supplementary material for: Haloimpatiens sporogenes sp. nov. and Haloimpatiens myeolchijeotgali sp. nov., anaerobic bacteria isolated from Myeolchi-jeot, a traditional Korean fermented anchovy
Source: J Microbiol. 2025 Jul 31;63(7):e2504009. doi: 10.71150/jm.2504009 (PMC13577056; doi:10.71150/jm.2504009)

Fig. S4. Two-dimensional thin-layer chromatoGrams showing the compositions of total polar lipids (A), glycolipids (B), aminolipids (C), and phospholipids (D) in strains FM7315^{T}. Total lipids were stained with 90% ethanolic molybdophosphoric acid, glycolipids with α-naphthol and sulfuric acid, amino lipids with ninhydrin, and phospholipids with both ninhydrin and the Dittmer-Lester reagent. Solvent system: (I) chloroform-methanol-water (65:25:4, v/v/v); (II) chloroform-acetic acid-methanol-water (80:15:12:4, v/v/v/v). DPG, diphosphatidylglycerol; PG, phosphatidylglycerol; APL, aminophospholipid; GL, unidentified glycolipid; UL, unidentified polar lipid.

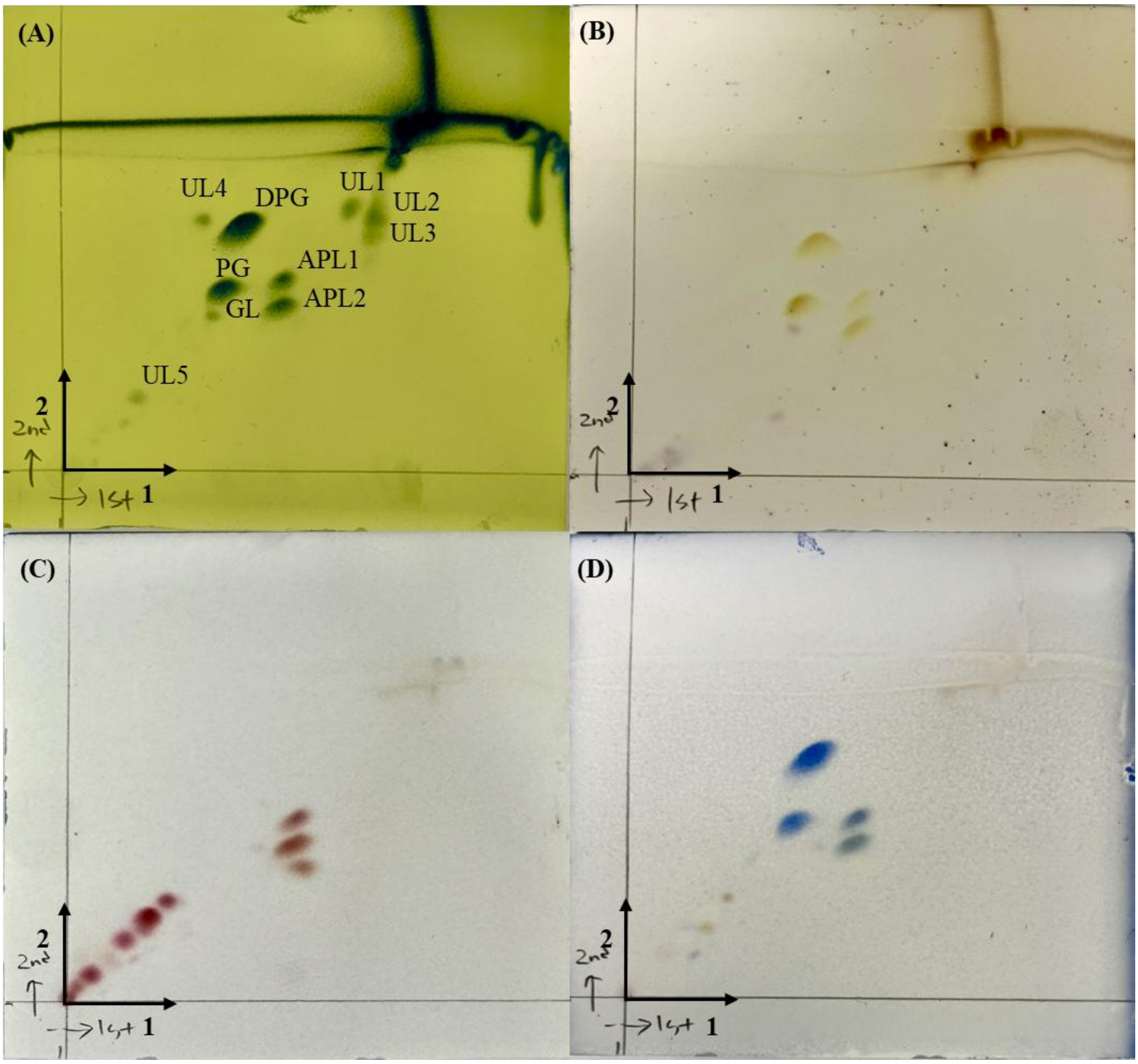

Supplement: Supplementary file 4 [file jm-2504009-Supplementary-Fig-S4.pdf]
