# Supplementary material for: Haloimpatiens sporogenes sp. nov. and Haloimpatiens myeolchijeotgali sp. nov., anaerobic bacteria isolated from Myeolchi-jeot, a traditional Korean fermented anchovy
Source: J Microbiol. 2025 Jul 31;63(7):e2504009. doi: 10.71150/jm.2504009 (PMC13577056; doi:10.71150/jm.2504009)
Supplement: Supplementary file 6 [file jm-2504009-Supplementary-Table-S1.pdf]

Table S1. Comparison of the genomic identities of strains FM7315^T and FM7330^T with those of their phylogenetically closely related species in the genus *Haloimpatiens* and the type species of the genus *Clostridium*, using AAI and POCP analysis. Strains: 1, *Haloimpatiens sporogenes* FM7315^T; 2, *Haloimpatiens myeolchijeotgali* FM7330^T; 3, *Haloimpatiens lingqiaonensis* KCTC 15321^T; 4, *Clostridium butyricum* DSM 10702^T

	**AAI or POCP value (%) for comparison genome of†:**			
**Strain**	**1**	**2**	**3**	**4**
**1**		68.08	71.55	59.46
**2**	53.92		70.10	59.22
**3**	54.55	57.83		59.84
**4**	32.73	39.38	35.93	

† Average amino acid identity (AAI) values are shown in the half of top right of the table; Percentage of conserved proteins (POCP) values are shown in the half of bottom left of the table.
