# Supplementary material for: Haloimpatiens sporogenes sp. nov. and Haloimpatiens myeolchijeotgali sp. nov., anaerobic bacteria isolated from Myeolchi-jeot, a traditional Korean fermented anchovy
Source: J Microbiol. 2025 Jul 31;63(7):e2504009. doi: 10.71150/jm.2504009 (PMC13577056; doi:10.71150/jm.2504009)
Supplement: Supplementary file 7 [file jm-2504009-Supplementary-Table-S2.pdf]

Table S2. Pairwise comparison of average nucleotide identity (ANI) and digital DNA-DNA hybridization (dDDH) values between strains FM7315^T and FM7330^T and the type strain of phylogenetically closely related species in the genus *Haloimpatiens*. Strains: 1, *Haloimpatiens sporogenes* FM7315^T; 2, *Haloimpatiens myeolchijeotgali* FM7330^T; 3, *Haloimpatiens lingqiaonensis* KCTC 15321^T

	**ANI or dDDH value (%) for comparison genome of†:**		
**Strain**	**1**	**2**	**3**
**1**		73.44	74.64
**2**	21.30		74.27
**3**	22.60	22.10	

† Average nucleotide identity (ANI) values are shown in the half of top right of the table; Digital DNA–DNA hybridization (dDDH) values are shown in the half of bottom left of the table.
