# Supplementary material for: Haloimpatiens sporogenes sp. nov. and Haloimpatiens myeolchijeotgali sp. nov., anaerobic bacteria isolated from Myeolchi-jeot, a traditional Korean fermented anchovy
Source: J Microbiol. 2025 Jul 31;63(7):e2504009. doi: 10.71150/jm.2504009 (PMC13577056; doi:10.71150/jm.2504009)
Supplement: Supplementary file 8 [file jm-2504009-Supplementary-Table-S3.pdf]

Table S3. Flagellar biosynthesis-related genes identified in the genomes of strains FM7315^T and FM7330^T

Gene	Function	Locus tag	
		**FM7315^T**	**FM7330^T**
FliE	Hook–basal body junction protein	ACER0A_RS07840	ACFIJ5_RS11575
FliA	Flagellar sigma factor (σ^{28})	ACER0A_RS07730	ACFIJ5_RS11465
FliF	MS ring (basal body) protein	ACER0A_RS07835	ACFIJ5_RS11570
FliG	C-ring protein; motor switch and torque generator	ACER0A_RS07830	ACFIJ5_RS11565
FliH	ATPase regulator in flagellar export	ACER0A_RS07825	ACFIJ5_RS11560
FliJ	Chaperone escort for protein export	ACER0A_RS07815	ACFIJ5_RS11550
FliK	Hook-length control protein	ACER0A_RS07810	ACFIJ5_RS11545
FliL	Motor-associated membrane protein	ACER0A_RS07775	ACFIJ5_RS11510
FliM	C-ring protein; rotation direction control	ACER0A_RS08010	ACFIJ5_RS11795
FliO	T3SS export apparatus component	ACER0A_RS07770	ACFIJ5_RS11505
FliP	T3SS export gate component	ACER0A_RS12380	ACFIJ5_RS11500
FliQ	T3SS export gate component	ACER0A_RS07760	ACFIJ5_RS11495
FliC	Flagellin; filament structural subuni	ACER0A_RS07945	ACFIJ5_RS11590
FliD	Filament cap protein	ACER0A_RS07955	ACFIJ5_RS11740
FliS	Flagellin chaperone	ACER0A_RS07960	ACFIJ5_RS11745
FlhA	T3SS export apparatus protein	ACER0A_RS07750	ACFIJ5_RS11485
FlgB	Proximal rod protein	ACER0A_RS07850	ACFIJ5_RS11590

FlgC	Rod structure protein	ACER0A_RS07845	ACFIJ5_RS11580
FlgD	Hook assembly initiator	ACER0A_RS07805	ACFIJ5_RS11540
FlgE	Hook protein	ACER0A_RS07795	ACFIJ5_RS11530
FlgK	Hook–filament junction protein	ACER0A_RS07990	ACFIJ5_RS11775
FlgL	Hook–filament junction protein	ACER0A_RS07985	ACFIJ5_RS11770
FlgM	Anti-σ^{28} factor	ACER0A_RS08000	ACFIJ5_RS11785
MotA	Stator protein (proton channel)	ACER0A_RS07785	ACFIJ5_RS11520
MotB	Stator anchoring protein	ACER0A_RS07780	ACFIJ5_RS11515
RpoD	Sigma factor (σ^{70}); general transcription	ACER0A_RS04465	ACFIJ5_RS12005
RpoN	Sigma factor (σ^{54}); flagellar gene activation	ACER0A_RS11255	ACFIJ5_RS03220
