# Supplementary material for: Haloimpatiens sporogenes sp. nov. and Haloimpatiens myeolchijeotgali sp. nov., anaerobic bacteria isolated from Myeolchi-jeot, a traditional Korean fermented anchovy
Source: J Microbiol. 2025 Jul 31;63(7):e2504009. doi: 10.71150/jm.2504009 (PMC13577056; doi:10.71150/jm.2504009)
Supplement: Supplementary file 9 [file jm-2504009-Supplementary-Table-S4.pdf]

Table S4. Comparison of cellular fatty acid compositions (%) between the two novel strains and *Haloimpatiens lingqiaonensis* KCTC 15321^T. Strains: 1, *H. sporogenes* FM7315^T, 2, FM7330^T, 3, *H. lingqiaonensis* KCTC 15321^T. The majority of fatty acid values (> 5%) are highlighted in bold. tr, trace element (< 1%); –, not detected

**Fatty acids**	**1**	**2**	**3**
**Saturated**			
$C_{10:0}$	tr	1.82	-
$C_{12:0}$	1.54	3.78	tr
$C_{13:0}$ iso	-	-	1.43
$C_{14:0}$	**5.56**	tr	**7.36**
$C_{15:0}$ 2OH	1.02	tr	**-**
$C_{15:0}$ iso	-	-	**12.34**
$C_{15:0}$ anteiso	tr	tr	**5.73**
$C_{16:0}$	**19.90**	**9.35**	**42.67**
$C_{17:0}$	1.23	tr	tr
$C_{17:0}$ 2OH	2.05	3.84	-
$C_{17:0}$ anteiso	1.04	tr	tr
$C_{18:0}$	2.74	tr	1.33
$C_{19:0}$	1.04	-	tr
$C_{19:0}$ cyc ω8c	1.15	-	-
$C_{19:0}$ iso	2.01	4.18	tr
**Unsaturated**			
$C_{15:1}$ ω8c	1.03	-	0.57
$C_{16:1}$ ω9c	4.00	-	2.96

$C_{17:1}$ anteiso A	1.29	-	-
$C_{17:1}$ ω8c	1.24	**8.29**	tr
$C_{18:1}$ ω7c 11-methyl	1.18	-	-
$C_{18:1}$ ω9c	**21.62**	**64.64**	1.3
$C_{20:1}$ ω7c	tr	1.31	tr
**Summed Feature**[†]			
1 ($C_{15:1}$ iso H/$C_{13:0}$ 3OH)	2.57	0.53	4.11
3 ($C_{16:1}$ ω7c/$C_{16:1}$ ω6c)	**6.34**	0.44	4.92
5 ($C_{18:0}$ anteiso/$C_{18:2}$ ω6,9c)	3.32	-	-
8 ($C_{18:1}$ ω7c/$C_{18:1}$ ω6c)	**9.38**	-	tr

[†]Two or three fatty acids could not be separated by the Microbial Identification System.
